# Famine Exposure in Early Life and Risk of Metabolic Syndrome in Adulthood: Comparisons of Different Metabolic Syndrome Definitions

**DOI:** 10.1155/2019/7954856

**Published:** 2019-12-06

**Authors:** Feng Ning, Jie Ren, Xin Song, Dong Zhang, Li Liu, Lei Zhang, Jianping Sun, Dongfeng Zhang, Zengchang Pang, Qing Qiao, on behalf of Qingdao Diabetes Prevention Program

**Affiliations:** ^1^Department of Epidemiology and Health Statistics, Qingdao University Medical College, 266021 Qingdao, China; ^2^Qingdao Centers for Disease Control and Prevention, 266033 Qingdao, China; ^3^Qingdao Institute of Preventive Medicine, 266033 Qingdao, China; ^4^Shandong Centers for Disease Control and Prevention, 250200 Jinan, China; ^5^Huangdao Centers for Disease Control and Prevention, 266000 Qingdao, China; ^6^Qingdao Endocrine and Diabetes Hospital, 266003 Qingdao, China; ^7^Weifang Medical College, 261000 Weifang, China; ^8^Department of Public Health, University of Helsinki, FI-00014 Helsinki, Finland

## Abstract

This study examined the association between famine exposure in early life and the risk of metabolic syndrome (MetS) in adulthood during the 1959–1961 Chinese Famine. Two cross-sectional surveys involving randomly selected Chinese adults aged 35–74 years in the Qingdao area were conducted. A total of 9,588 individuals were grouped into four birth cohorts of unexposed (born between January 1, 1962, and December 31, 1975), fetal-exposed (born between January 1, 1959, and December 31, 1961), childhood-exposed (born between January 1, 1949, and December 31, 1958), and adolescence/adult-exposed cohorts (born between January 1, 1931, and December 31, 1948). We assessed the prevalence rate of MetS in relation to famine exposure according to three definitions of MetS by the National Cholesterol Education Program Adult Treatment Panel III (NCEP-ATP III), International Diabetes Federation (IDF), and China Diabetes Society (CDS). According to the CDS criterion, the prevalence rates of MetS were 17.8%, 25.7%, 31.1%, and 45.3% in the unexposed, fetal-, childhood-, and adolescence/adult-exposed cohorts, respectively (*P* < 0.001). For the CDS criteria, compared with individuals without famine exposure, odds ratios (95% confidence interval) for MetS were 1.36 (1.02–1.81), 1.36 (1.06–1.75), and 1.60 (1.06–2.41) in women and 1.10 (0.79–1.53), 1.07 (0.79–1.42), and 1.21 (0.74–1.99) in men who were exposed in the fetal, childhood, and adolescence/adult periods, respectively, after adjustment for age, study cohorts, residential areas, education levels, income levels, current smoking, and current drinking. The same trend was observed in fetal and childhood exposure for the NCEP-ATP III and IDF definitions, except for a marginal effect in adolescence/adult exposure. Sensitivity analysis revealed that the odds ratios for MetS prevalence for the CDS definition were 1.37 (1.03–1.82), 1.40 (1.09–1.79), and 1.58 (1.04–2.40) among fetal, childhood, and adolescence/adult exposure in rural areas, respectively. The CDS definition is superior to the other definitions for determining the association between famine exposure and MetS with respect to early life. Famine exposure in early life is associated with an increased risk of MetS in later life, especially in women. Early-life malnutrition and later life overnutrition were critical in determining adulthood metabolic disorders.

## 1. Introduction

Metabolic syndrome (MetS) is characterized as insulin resistance and comprises obesity, hypertension, glucose intolerance and dyslipidemia, and increased risk of type 2 diabetes, cardiovascular diseases and other chronic diseases [1, 2]. With urbanization and Westernization, MetS prevalence and its related risk factors have sharply increased in China [3]. Uteromalnutrition and DNA methylation have been theorized to be the origins of metabolic diseases in later life [4–6]. In addition, an abnormal birth weight, low socioeconomic status during infancy, and catch-up growth increase the likelihood of adulthood metabolic disorders and chronic diseases [7, 8]. Several studies have determined that famine exposure in infants may be associated with obesity [9], hypertension [10], diabetes [11], and MetS [12, 13] in adulthood. However, some research groups have developed ethnicity-specific MetS criteria, resulting in findings on the famine effect that diverge from previous studies [12–15]. Thus far, no population-based study had compared the famine exposure effect on MetS by definitions from the National Cholesterol Education Program Adult Treatment Panel III (NCEP-ATP III), International Diabetes Federation (IDF), and China Diabetes Society (CDS). Thus, in the current study, we conducted two consecutive population-based surveys exposed to the 1959–1961 Chinese Famine during early life, to compare associations between famine exposure and the risk of MetS based on three criteria in adult life.

## 2. Materials and Methods

The participants and protocol of our survey have been described in our previously published reports [16, 17]. Briefly, a stratified, random cluster sampling method was used in 2006 and 2009 to select from a general population aged 35–74 years in Qingdao, China. Three urban and three rural areas were randomly selected according to their socioeconomic characteristics. A total of 5,355 participants underwent face-to-face interviews in the 2006 survey, and 5,165 more individuals were included in the 2009 survey; response rates were 87.8% and 86.1%, respectively. Considering the survey protocol and laboratory test that had been standardized in two cross-sectional studies, we combined those two studies in the data analysis. After excluding individuals with missing data, 9,588 participants were included in the current study. The ethics committees of the Qingdao Health Bureau and Qingdao Centers for Disease Control and Prevention approved the surveys. Written informed consent was obtained from each participant. The clinical trial was registered (registration no. NCT01053195) and is detailed in https://clinicaltrials.gov/ct2/home.

### 2.1. Famine Exposure Cohorts

The 1959–1961 Chinese Famine, dubbed as the “3-year natural disaster” in Chinese literature, has had long-term and nationwide health implications in China [18]. In this study, individuals exposed to the 1959–1961 Chinese Famine were, according to their age at exposure, categorized into three cohorts of fetal exposure (born between January 1, 1959, and December 31, 1961), childhood exposure (born between January 1, 1949, and December 31, 1958), and adolescent/adult exposure (born between January 1, 1931, and December 31, 1948). Participants born after January 1, 1962, were defined as the unexposed cohort (born between January 1, 1962, and July 1, 1975).

### 2.2. Definitions of MetS

We applied three MetS definitions in the current study. The NCEP-ATP III criterion [19], used as our definition of MetS, defines a person as having MetS if they meet three or more of the following criteria: (1) a fasting triglyceride level of ≥1.69 mmol/L; (2) a high-density lipoprotein cholesterol (HDL-C) of <1.04 mmol/L for men and <1.29 mmol/L for women; (3) a fasting plasma glucose(FPG) of ≥5.6 mmol/L, the receiving of diabetes treatment, or a previous diagnosis of diabetes; (4) a waist circumference of ≥102 cm for men and ≥88 cm for women; and (5) a systolic blood pressure (SBP) of ≥130 mmHg, a diastolic blood pressure (DBP) of ≥85 mmHg, or the receiving of hypertension treatment.

According to the IDF definition updated in 2006 [20], an individual has MetS if he or she has central obesity (defined as a waist circumference of ≥90 cm and ≥80 cm for Chinese men and women, respectively), in addition to any two of the following components: (1) a serum triglyceride level of ≥1.7 mmol/L; (2) a HDL-C of <1.04 mmol/L for men and <1.29 mmol/L for women; (3) a SBP of >130 mmHg, a DBP of >85 mmHg, or treatment of previously diagnosed hypertension; and (4) a FPG of >5.6 mmol/L or previously diagnosed diabetes.

We also assessed MetS prevalence according to the CDS 2017 criterion, where a person has MetS if he or she meets three or more of the following criteria: (1) a waist circumference of ≥90 cm for men and ≥85 cm for women; (2) a FPG of ≥6.1 mmol/L, a 2 h plasma glucose (2hPG) of ≥7.8 mmol/L, or previously diagnosed diabetes; (3) a SBP of ≥130 mmHg, a DBP of ≥85 mmHg, or previously diagnosed hypertension; (4) a fasting triglyceride level of ≥1.7 mmol/L; and (5) a HDL-C of <1.04 mmol/L [21].

### 2.3. Study Parameters

After all individuals fasted overnight for at least 10 h, blood samples were collected from 7:00 to 9:30 am to determine fasting blood glucose levels. Individuals without a history of diabetes underwent a 75 g oral glucose tolerance test, and a second blood sample was obtained at 2 h after ingestion of 75 g glucose. Blood samples were collected from the antecubital vein into EDTA tubes containing sodium fluoride, which were centrifuged at the survey site. The samples were placed in ice-cooled containers and transported immediately into Qingdao Hiser Hospital in 2006 and Qingdao Endocrine and Diabetes Hospital in 2009 for laboratory testing. Plasma glucose parameters were determined using the glucose oxidase method (Olympus-AU, Olympus Co., Tokyo, Japan). Total cholesterol and serum triglycerides were determined using enzymatic techniques, and HDL-C was measured using a direct method with an Olympus reagent with OLYMPUS-AU640 Automatic Analyzers both in the 2006 and 2009 surveys (Olympus Optical, Olympus Co., Tokyo, Japan).

Height and weight were measured with the individuals wearing light clothes but no shoes. Waist circumference was measured at the minimal abdominal girth between the rib cage and the iliac crest. Body mass index (BMI) was calculated as weight in kilograms divided by the square of height in meters. Three consecutive blood pressure readings, at least 1 min apart, were taken from the right arm of seated individuals, and the average of the three readings was recorded. A positive family history of diabetes was defined as having at least one diabetic family member among first-degree relatives. Family income was categorized into low (≤1799 Renminbi (RMB) monthly), medium (1800–2999 RMB monthly), and high (≥3000 RMB monthly). An individual's total score on the Chinese version of the Zung self-reported depression scale was used as an indicator of depression. A cut-off score of 40 indicated positive depression [22].

### 2.4. Statistical Analyses

Continuous variables are presented as their mean (standard error), and categorical variables as their number (percentage). We used a general linear model, adjusted for age, to compare the differences among the means of the four cohorts. Categorical data were analyzed using the *χ*^2^ test. *Z* score transformations of the lipid and glucose parameters were employed in the data analysis to reduce the methodological differences between studies. Logistic regression analysis was used to estimate the odds ratios (ORs) and 95% confidence intervals (CIs) to determine the relationship between famine exposure and MetS in later life, after adjustment for age, sex, study cohorts, residential areas, education levels, income levels, current smoking, and current drinking. Interactions between family history of diabetes, depression, residential areas, income levels, education levels, current smoking, and current drinking on MetS were tested using a multiplicative factor in the multivariable logistic regression models. Data were analyzed using SPSS, version 22.0 (SPSS IBM company, Chicago, IL). Two-sided *P* values of 0.05 were considered statistically significant.

## 3. Results

**Figure 1:**
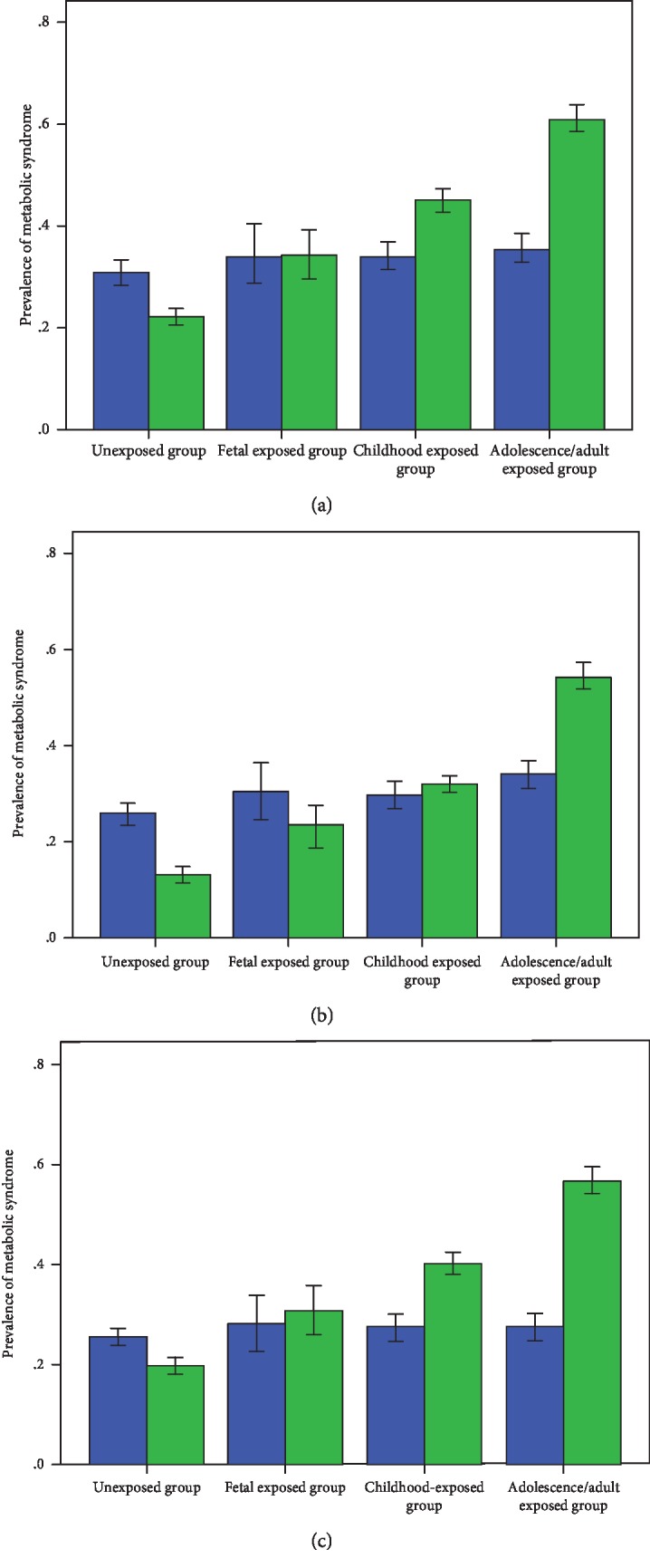
Sex-specific prevalence (95% confidence interval) of metabolic syndrome in different life stages exposed to the 1959-1961 Chinese Famine, according to the NCEP-ATP III (a), CDS (b), IDF (c) diagnosed criteria. The blue bar indicates men and green bar for women.

Of the total 9,588 participants, 2,380 men and 3,553 women were exposed to the Chinese Famine in the current study. As detailed in Table 1, the mean values for age, waist circumference, FPG, HDL-C, total cholesterol, and serum triglycerides were greater in men than those in women, whereas BMI and 2hPG values were greater in women than in men (*P* < 0.01 for all comparisons). According to Table 2, compared with unexposed individuals, those with famine exposure were older, more obese, and had greater mean values of FPG, 2hPG, and DBP across the three MetS definitions (*P* < 0.01 for all comparisons). Based on the CDS definition, the prevalence rates of MetS were 17.8%, 25.7%, 31.1%, and 45.3% in the unexposed, fetal-, childhood-, and adolescence/adult-exposed cohorts, respectively (*P* < 0.01 for all comparisons). The sex-specific prevalences and 95% confidence interval of MetS among the individuals of the exposed cohorts are detailed in Figures 1(a)–1(c). We observed a risk gradient between famine exposure and MetS prevalence for all three definitions, particularly in women.

Compared with the unexposed group, the ORs for MetS, for the NCEP-ATP III and CDS criteria, were significantly higher in the fetal- and childhood-exposed cohorts. However, ORs for adolescence/adult-exposed cohorts were significant only for the CDS criterion and marginal for the other criteria (see Table 3). In the multivariable model, compared with the unexposed cohort, ORs (95% CIs) for the prevalence of MetS for the CDS definition were 1.29 (1.04–1.60), 1.31 (1.10–1.60), and 1.62 (1.19–2.21) in the fetal, childhood, and adolescent/adult famine-exposed cohorts, respectively. We also observed an association between a lower socioeconomic status and MetS prevalence, independent of an individual's early life exposure to famine. In rural areas, the ORs for MetS prevalence for the CDS definition were 1.37 (1.03–1.82), 1.40 (1.09–1.79), and 1.58 (1.04–2.40) in the three exposed cohorts, respectively. In urban areas, the corresponding figures were 1.09 (0.78–1.52), 1.18 (0.88–1.58), and 1.40 (0.87–2.26), respectively (detailed in Supplement [Supplementary-material supplementary-material-1]). Similar trends were observed for the other two MetS definitions. However, no significant interactions between famine exposure and residential areas were discovered for both men and women—the corresponding figures were 0.76 (0.54–1.06) and 0.89 (0.66–1.19), respectively. Additionally, no significant interactions were observed between famine exposure and depression, income level, education level, current smoking, current drinking, and a family history of diabetes in both men and women.

As detailed in Tables 4–6, for women, the association between famine exposure and MetS risk was significant in the CDS and IDF definitions but moderate in the NCEP-ATP III definition. This association was nonsignificant for men for all three definitions. For the CDS definition and relative to the unexposed group, the corresponding ORs and 95% CIs for MetS prevalence were 1.36 (1.02–1.81), 1.36 (1.06–1.75), and 1.60 (1.06–2.41) in the three groups, respectively. The other two definitions had similar trends.

Although we lacked a matched age range of individuals unexposed to the 1959–1961 Chinese Famine, we additionally adjusted for age as covariates in the exposure of age intervals in a multivariable model. As detailed in Supplement Tables [Supplementary-material supplementary-material-1], the results did not lead to substantial changes in the trends for the 3-year interval age groups, after additional adjustment for age using the three different MetS definitions.

## 4. Discussion

This study demonstrated a significant association between famine exposure (to the 1959–1961 Chinese Famine) during early life and MetS prevalence in adult women, but not in men. Thus, early malnutrition identification and intervention prevents metabolic disorders in later life.

Several studies have investigated the relationship between famine exposure in early life and adulthood MetS [23–26]. A recent meta-analysis comprising 25,649 individuals determined that fetal famine exposure (to the 1959–1961 Chinese Famine) resulted in a moderate risk of MetS (OR = 1.11; 95% CI: 1.00–1.22) relative to pre- and postfamine exposure groups [23]. Moreover, studies that have used data from the China SPECT and 2002 China National Nutrition and Health Survey (CHNS) have determined fetal exposure to be significantly associated with MetS risk in later life, as defined by the IDF [12], NCEP-ATP III [13], and CDS [24]. Our study further confirmed the strong relationship of fetal and childhood exposure with MetS risk for all three definitions, and adolescent/adult exposure only for the CDS definitions. It has been suggested that the fetal and childhood periods are when individuals are prone to be affected by malnutrition, which accelerates metabolic disorder's progression in adulthood. However, such positive findings were not obtained in European studies such as the Dutch Winter Famine Study and the Siege of Leningrad Study [15, 25, 26]. The Dutch Winter Families study found that individuals exposed to famine during World War II were more insulin resistant than those unexposed during their fetal period [25]. These inconsistent results have been attributed to famine duration, ethnic differences, and the recent rapid economic growth of China.

These associations between famine exposure and MetS are stronger among individuals who have a Western dietary pattern or who lived in a rural area in adulthood. Using data from the 2002 CHNS, a study determined that only fetal exposure to severe famine was related to an affluent or western dietary pattern in later life, which may result in a high MetS risk [14]. Moreover, according to a study that used the China SPECT, in rural areas, exposure throughout both the fetal and childhood periods increased the risk for type 2 diabetes in adulthood [27]. Consistent with a higher prevalence of type 2 diabetes and MetS in the rural areas of Qingdao during the past 10 years [17], we observed that exposure to urbanization intensified the association between fetal famine exposure and adulthood metabolic disorders. However, the interactions between famine exposure and residential areas on MetS were not observed across the three definitions in our study.

In our sex-specific analysis, we observed that the association between famine exposure and MetS prevalence was significantly stronger in women, but not in men. A similar trend has been observed in several studies based on the 1959–1961 Chinese Famine [13, 28, 29]. A recent meta-analysis indicated a significant association between overweight and obesity risk in women who were exposed to malnutrition in early life, but not in men [30]. This gender difference has several explanations. First, survivor bias in and the cultural particularities of China may explain why boys who survived the famine period were more likely to receive sufficient nutrition relative to girls. Second, several studies have indicated that biological differences in sex hormones, body composition, and glucose metabolism may contribute to the famine effect. This gender disparity in the famine effect requires further investigation.

Explanations for the effect of famine exposure on metabolic disorders in later life have also emerged [31–35]. Malnutrition during the critical growth stages may lead to early adaptations in body structure and function, such as the establishment of a thrifty phenotype, which is beneficial to most organs in the short term [4, 6]. The common soil hypothesis also suggests that early malnutrition and relative overnutrition in later life result in the development of MetS. Moreover, epigenetic modulation as a result of early-life malnutrition may promote an adverse metabolic phenotype in later life [5]. The Dutch Winter Families study suggested that DNA methylation may be a common consequence of prenatal famine exposure and that these changes depend on gender exposure and gestational timing [34]. A meta-analysis comprising 16,601 Europeans discovered that a low birth weight was associated with the incidence of type 2 diabetes [35]. In a subgroup that was formed by segmenting birth-weight data (*N* = 1,344), we found no association between birth weight and MetS prevalence in the current study. The mechanism between early life famine exposure and the development of metabolic disorders requires further investigation.

Our study has some strengths. First, the random representative population-based sample was large and composed of both urban and rural residents. Second, all interviews were conducted face to face by trained health workers, and the anthropometric parameters were measured on site, which allowed for rigorous quality control. The current study also has some limitations. First, because of the cross-sectional study design, the causal relation between famine exposure and MetS risk cannot be inferred. Second, birth weight was a potential confounder but its data for all participants were not available. Nonetheless, we assessed an association between birth weight and MetS prevalence in a subgroup analysis and observed no significant association. The effect of birth weight, however, requires further investigation. Third, age was strongly associated with MetS prevalence and recognized as a potential confounder based on our current findings. Because the 1959–1961 Chinese Famine affected almost everyone living in Qingdao when it occurred, the famine-exposed age group cannot be matched to its unexposed counterparts. Fourth, a classification bias may have occurred because metabolic components increase with age. Nonetheless, we used a 3-year interval for the age groups to reduce the age effect on MetS prevalence. The associations between famine exposure and MetS risk were robust in a sensitivity analysis independent of age.

## 5. Conclusion

Famine exposure in early life was associated with an increased risk of MetS in adulthood, especially in women and those living in rural areas. The CDS definition was superior to these definitions for predicting the association between famine exposure and MetS for all early life periods. Early-life malnutrition and later life overnutrition showed to be critical for metabolic disorders in adult life.

## Figures and Tables

**Table 1:** Baseline characteristics of study population.

	Men	Women	Total	*P* value
No.	3782	5806	9588	
Age (years)	51.8 (10.9)	51.0 (10.3)	51.3 (10.6)	0.00
BMI (kg/m^2^)	25.2 (3.4)	25.6 (3.7)	25.4 (3.6)	0.00
Obesity (%)	22.7	23.3	24.2	0.01
Waist circumference (cm)	86.5 (10.3)	82.8 (10.1)	84.3 (10.3)	0.00
SBP (mmHg)	134.5 (20.2)	133.0 (22.9)	133.6 (21.9)	0.00
DBP (mmHg)	85.4 (12.2)	83.0 (11.9)	84.0 (12.1)	0.00
FPG (mmol/L)	0.04 (1.00)	-0.02 (1.00)	NA	0.00
2hPG (mmol/L)	-0.04 (1.02)	0.03 (0.98)	NA	0.00
Family history of diabetes (%)	12.9	15.8	14.7	0.01
Total cholesterol (mmol/L)	-0.01 (0.99)	0.01 (1.01)	NA	0.44
HDL-C (mmol/L)	-0.05 (1.03)	0.04 (0.98)	NA	0.00
Triglycerides (mmol/L)	0.07 (1.16)	-0.05 (0.88)	NA	0.00
Family income (%)				
Low	61.0	78.2	71.4	0.00
Medium	24.2	17.6	20.2	
High	14.8	4.2	8.4	
Education levels (%)				
Primary	26.6	38.5	33.8	0.00
Secondary	39.1	34.0	36.0	
Advanced	34.2	27.5	30.2	
Current smoking (%)	52.3	2.7	22.3	0.00
Current drinking (%)	42.9	1.4	17.8	0.17

Data are presented as mean and standard deviation for continuous variables or percentage for categorical variables. *P* value in *t*-test for means or *χ*^2^ test for proportion differences between men and women. BMI: body mass index; DBP: diastolic blood pressure; FPG: fasting plasma glucose; HDL-C: high-density lipoprotein cholesterol; 2hPG: 2-hour plasma glucose; SBP: systolic blood pressure.

**Table 2:** Baseline characteristics of study population according to Chinese famine exposure between 1959 and 1961.

	Unexposed group	Fetal-exposed group	Childhood-exposed group	Adolescence/adult-exposed group	*P* value
No.	3655	621	2877	2435	
Age (years)	40.8 (0.06)	47.3 (0.09)	53.5 (0.07)	65.6 (0.09)	0.01
Men (%)	38.4	39.1	36.7	44.4	0.01
Rural area (%)	32.6	41.9	42.4	41.2	0.01
BMI (kg/m^2^)	24.6 (0.12)	25.3 (0.15)	25.7 (0.07)	26.3 (0.15)	0.01
Obesity (%)	20.2	22.7	23.3	24.2	0.01
Waist circumference (cm)					
Men	85.0 (0.56)	87.2 (0.71)	86.5 (0.33)	88.3 (0.69)	0.01
Women	82.1 (0.39)	82.8 (0.52)	83.2 (0.25)	83.6 (0.55)	0.22
SBP (mmHg)	131.3 (0.65)	132.6 (0.84)	133.8 (0.39)	137.0 (0.86)	0.01
DBP (mmHg)	80.8 (0.38)	84.0 (0.50)	85.8 (0.23)	86.6 (0.51)	0.01
FPG (mmol/L)	-0.06 (0.03)	0.02 (0.04)	0.01 (0.02)	0.07 (0.04)	0.10
2hPG (mmol/L)	-0.15 (0.03)	-0.04 (0.04)	0.02 (0.02)	0.22 (0.05)	0.01
Total cholesterol (mmol/L)	-0.07 (0.03)	0.01 (0.04)	0.09 (0.02)	0.00 (0.04)	0.01
HDL-C (mmol/L)	-0.02 (0.03)	0.03 (0.04)	0.06 (0.02)	-0.05 (0.04)	0.01
Triglycerides (mmol/L)	0.08 (0.03)	0.07 (0.04)	0.00 (0.02)	-0.13 (0.04)	0.01
Family income (%)					
Low	66.7	71.8	72.3	77.3	0.01
Medium	22.4	19.3	21.3	16.0	
High	10.9	8.9	6.5	6.7	
Education levels (%)					
Primary	16.6	19.3	36.4	60.5	0.01
Secondary	46.4	34.5	36.6	20.0	
Advanced	37.0	46.2	27.0	19.5	
Current smoking (%)	22.4	23.5	21.8	22.1	0.80
Current drinking (%)	18.0	20.8	17.6	17.0	0.17

Data are age-adjusted mean (SE) or as noted. BMI: body mass index; DBP: diastolic blood pressure; FPG: fasting plasma glucose; HDL-C: high-density lipoprotein cholesterol; 2hPG: 2-hour plasma glucose; SBP: systolic blood pressure.

**Table 3:** Odds ratio (95% confidence interval) of metabolic syndrome in relation to different life stages at famine exposure.

Exposure stages	Prevalence of MetS (%)	Model 1	Model 2
NCEP-ATP III criterion			
Unexposed	25.4	1.00 (Ref)	1.00 (Ref)
Fetal-exposed	34.3	1.25 (1.03-1.52)	1.22 (1.00-1.49)
Childhood-exposed	41.1	1.37 (1.15-1.62)	1.30 (1.08-1.58)
Adolescence/adult-exposed	49.8	1.35 (1.01-1.80)	1.25 (0.89-1.73)
CDS criterion			
Unexposed	17.8	1.00 (Ref)	1.00 (Ref)
Fetal-exposed	25.7	1.29 (1.04-1.59)	1.29 (1.04-1.60)
Childhood-exposed	31.1	1.36 (1.13-1.64)	1.31 (1.10-1.60)
Adolescence/adult-exposed	45.3	1.67 (1.23-2.26)	1.62 (1.19-2.21)
IDF criterion			
Unexposed	21.8	1.00 (Ref)	1.00 (Ref)
Fetal-exposed	29.5	1.21 (0.99-1.48)	1.23 (1.00-1.52)
Childhood-exposed	35.4	1.26 (1.06-1.51)	1.27 (1.04-1.55)
Adolescence/adult-exposed	43.5	1.21 (0.90-1.63)	1.23 (0.88-1.72)

Model 1: adjusted for age and sex. Model 2: adjusted for age, study cohort, residential area, sex, education levels, income levels, current smoking, and current drinking.

**Table 4:** Odds ratio (95% confidence interval) of metabolic syndrome in relation to different life stages at famine exposure by the NCEP-ATP III definition.

	Univariate	Model 1	Model 2	
Men				
Unexposed	1 (Ref)	1 (Ref)	1 (Ref)	
Fetal-exposed	1.17 (0.87-1.56)	1.19 (0.87-1.62)	1.03 (0.74-1.43)	
Childhood-exposed	1.16 (0.98-1.38)	1.21 (0.91-1.60)	1.07 (0.78-1.46)	
Adolescence/adult-exposed	1.24 (1.05-1.47)	1.34 (0.84-1.60)	1.09 (0.64-1.85)	
Women				
Unexposed	1 (Ref)	1 (Ref)	1 (Ref)	
Fetal-exposed	1.85 (1.46-2.34)	1.25 (0.97-1.60)	1.27 (0.98-1.65)	
Childhood-exposed	2.91 (2.54-3.33)	1.34 (1.07-1.68)	1.33 (1.04-1.71)	
Adolescence/adult-exposed	5.55 (4.79-6.44)	1.22 (0.84-1.78)	1.22 (0.80-1.87)	

Model 1: adjusted for age. Model 2: adjusted for age, study cohort, residential area, sex, education levels, income levels, current smoking, and current drinking.

**Table 5:** Odds ratio (95% confidence interval) of metabolic syndrome in relation to different life stages at famine exposure by the CDS definition.

	Univariate	Model 1	Model 2	
Men				
Unexposed	1 (Ref)	1 (Ref)	1 (Ref)	
Fetal-exposed	1.27 (0.94-1.71)	1.27 (0.92-1.75)	1.10 (0.79-1.53)	
Childhood-exposed	1.22 (1.02-1.45)	1.22 (0.92-1.64)	1.07 (0.79-1.42)	
Adolescence/adult-exposed	1.48 (1.25-1.77)	1.51 (0.93-2.44)	1.21 (0.74-1.99)	
Women				
Unexposed	1 (Ref)	1 (Ref)	1 (Ref)	
Fetal-exposed	1.97 (1.51-2.59)	1.31 (0.98-1.74)	1.36 (1.02-1.81)	
Childhood-exposed	3.16 (2.70-3.70)	1.40 (1.09-1.79)	1.36 (1.06-1.75)	
Adolescence/adult-exposed	8.00 (6.80-9.42)	1.62 (1.08-2.42)	1.60 (1.06-2.41)	

Model 1: adjusted for age. Model 2: adjusted for age, study cohort, residential area, sex, education levels, income levels, current smoking, and current drinking.

**Table 6:** Odds ratio (95% confidence interval) of metabolic syndrome in relation to different life stages at famine exposure by the IDF definition.

	Univariate	Model 1	Model 2
Men			
Unexposed	1 (Ref)	1 (Ref)	1 (Ref)
Fetal-exposed	1.15 (0.85-1.56)	1.12 (0.81-1.56)	1.01 (0.71-1.43)
Childhood-exposed	1.11 (0.92-1.33)	1.05 (0.78-1.42)	0.96 (0.69-1.33)
Adolescence/adult-exposed	1.11 (0.93-1.33)	1.01 (0.61-1.66)	0.85 (0.49-1.49)
Women			
Unexposed	1 (Ref)	1 (Ref)	1 (Ref)
Fetal-exposed	1.78 (1.40-2.27)	1.22 (0.94-1.57)	1.31 (1.01-1.72)
Childhood-exposed	2.73 (2.38-3.14)	1.29 (1.02-1.61)	1.37 (1.06-1.76)
Adolescence/adult-exposed	5.30 (4.56-6.15)	1.21 (0.83-1.78)	1.38 (0.90-2.11)

Model 1: adjusted for age. Model 2: adjusted for age, study cohort, residential area, sex, education levels, income levels, current smoking, and current drinking.

## Data Availability

The survey data used to support the findings of this study have not been made available because the data ownership belongs to the third party.

## Supplementary Materials

Supplementary Materials Supplement table 1: odds ratio (95% confidence interval) of metabolic syndrome in relation to different life stages and age group at famine exposure by the NCEP-ATP III metabolic syndrome definition. Supplement table 2: odds ratio (95% confidence interval) of metabolic syndrome in relation to different life stages and age group at famine exposure by the CDS metabolic syndrome definition. Supplement table 3: odds ratio (95% confidence interval) of metabolic syndrome in relation to different life stages and age group at famine exposure by the IDF metabolic syndrome definition. Supplement table 4: odds ratio (95% confidence interval) of metabolic syndrome in relation to different life stages at famine exposure in urban and rural areas.
