# Supplementary material for: Famine Exposure in Early Life and Risk of Metabolic Syndrome in Adulthood: Comparisons of Different Metabolic Syndrome Definitions
Source: J Diabetes Res. 2019 Dec 6;2019:7954856. doi: 10.1155/2019/7954856 (PMC6925820; doi:10.1155/2019/7954856)
Supplement: Supplementary Materials — Supplement table 1: odds ratio (95% confidence interval) of metabolic syndrome in relation to different life stages and age group at famine exposure by the NCEP-ATP III metabolic syndrome definition. Supplement table 2: odds ratio (95% confidence interval) of metabolic syndrome in relation to different life stages and age group at famine exposure by the CDS metabolic syndrome definition. Supplement table 3: odds ratio (95% confidence interval) of metabolic syndrome in relation to different life stages and age group at famine exposure by the IDF metabolic syndrome definition. Supplement table 4: odds ratio (95% confidence interval) of metabolic syndrome in relation to different life stages at famine exposure in urban and rural areas. [file 7954856.f1.pdf]

Supplement table 1. Odds ratio (95% confidence interval) of metabolic syndrome in relation to different life stages and age group at famine exposure by the NCEP-ATPIII metabolic syndrome definition.

	Univariate	Model1	Model 2	Model 3
Non-exposed(1971-1975)	0.62(0.50-0.77)	0.70(0.54-0.89)	0.66(0.49-0.88)	0.60(0.48-0.75)
Non-exposed(1968-1970)	0.69(0.57-0.84)	0.75(0.60-0.93)	0.74(0.59-0.94)	0.71(0.58-0.86)
Non-exposed(1965-1967)	0.72(0.59-0.88)	0.75(0.61-0.92)	0.76(0.62-0.94)	0.74(0.60-0.91)
Non-exposed(1962-1964)	1(Ref)	1(Ref)	1(Ref)	1(Ref)
Fetal-exposed(1959-1961)	1.20(0.98-1.47)	1.15(0.94-1.42)	1.19(0.96-1.48)	1.23(1.00-1.51)
Childhood-exposed(1956-1958)	1.23(1.03-1.48)	1.14(0.94-1.39)	1.19(0.96-1.48)	1.25(1.05-1.50)
Childhood-exposed(1954-1956)	1.53(1.28-1.83)	1.36(1.09-1.69)	1.44(1.12-1.86)	1.56(1.30-1.88)
Childhood-exposed(1951-1953)	2.18(1.86-2.56)	1.82(1.41-2.35)	2.00(1.46-2.72)	2.25(1.90-2.66)
Adolescence/Adult exposed(1931-1950)	2.27(1.97-2.64)	1.68(1.17-2.41)	1.99(1.24-3.17)	2.42(2.06-2.85)

Model: Adjusted for age and sex.

Model 2: Adjusted for age, cohort, residential area, sex, education levels, income levels, current smoking and current drinking.

Model 3: Removed age from model 2.

Supplement table 2. Odds ratio (95% confidence interval) of metabolic syndrome in relation to different life stages and age group at famine exposure by the CDS metabolic syndrome definition.

	**Univariate**	**Model1**	**Model 2**	**Model 3**
Non-exposed(1971-1975)	0.63(0.49-0.80)	0.74(0.56-0.97)	0.66(0.62-0.78)	0.71(0.52-0.97)
Non-exposed(1968-1970)	0.72(0.58-0.89)	0.74(0.56-0.97)	0.75(0.58-0.97)	0.80(0.62-1.03)
Non-exposed(1965-1967)	0.63(0.50-0.80)	0.67(0.53-0.85)	0.65(0.51-0.83)	0.68(0.53-0.86)
Non-exposed(1962-1964)	1(Ref)	1(Ref)	1(Ref)	
Fetal-exposed(1959-1961)	1.23(0.98-1.54)	1.17(0.93-1.47)	1.28(1.02-1.61)	1.19(0.94-1.50)
Childhood-exposed(1956-1958)	1.28(1.05-1.55)	1.14(0.92-1.42)	1.23(0.98-1.55)	1.15(0.92-1.45)
Childhood-exposed(1954-1956)	1.51 (1.24-1.84)	1.28(1.01-1.62)	1.16(0.89-1.52)	1.27(0.97-1.66)
Childhood-exposed(1951-1953)	2.23(1.88-2.65)	1.72(1.32-2.25)	1.64(1.19-2.26)	1.74(1.27-2.40)
Adolescence/Adult exposed(1931-1950)	3.02(2.57-3.54)	1.97(1.35-2.87)	1.79(1.11-2.89)	2.05(1.27-3.30)

Model: Adjusted for age and sex.

Model 2: Adjusted for age, cohort, residential area, sex, education levels, income levels, current smoking and current drinking.

Model 3: Removed age from model 2.

Supplement table 3. Odds ratio (95% confidence interval) of metabolic syndrome in relation to different life stages and age group at famine exposure by the IDF metabolic syndrome definition.

	**Univariate**	**Model1**	**Model2**	**Model 3**
Non-exposed(1971-1975)	0.62(0.49-0.78)	0.72(0.55-0.93)	0.61(0.45-0.82)	0.59(0.47-0.75)
Non-exposed(1968-1970)	0.71(0.58-0.87)	0.79(0.63-0.98)	0.72(0.57-0.92)	0.71(0.57-0.87)
Non-exposed(1965-1967)	0.69(0.59-0.86)	0.73(0.59-0.91)	0.70(0.56-0.88)	0.69(0.56-0.86)
Non-exposed(1962-1964)	1(Ref)	1(Ref)	1(Ref)	
Fetal-exposed(1959-1961)	1.17(0.94-1.45)	1.14(0.90-1.39)	1.19(0.95-1.49)	1.21(0.97-1.50)
Childhood-exposed(1956-1958)	1.19(0.97-1.43)	1.08(0.88-1.32)	1.17(0.94-1.47)	1.20(0.99-1.45)
Childhood-exposed(1954-1956)	1.44(1.20-1.74)	1.24(0.99-1.56)	1.39(1.07-1.81)	1.44(1.19-1.74)
Childhood-exposed(1951-1953)	2.05(1.74-2.41)	1.62(1.25-2.10)	1.96(1.43-2.70)	2.06(1.74-2.45)
Adolescence/Adult exposed(1931-1950)	2.18(1.87-2.54)	1.47(1.02-2.13)	2.10(1.30-3.37)	2.28(1.92-2.69)

Model1 : Adjusted for age and sex.

Model 2: Adjusted for age, cohort, residential area, sex, education levels, income levels, current smoking and current drinking.

Model 3: Removed age from model 2.

Supplement table 4.Odds ratio (95% confidence interval) of metabolic syndrome in relation to different life stages at famine exposure in urban and rural areas.

Exposure stages	Prevalence of metabolic syndrome (%)	Urban areas	Prevalence of metabolic syndrome (%)	Rural areas
**ATP III criteria**				
Non-exposed	27.8	1.00(Ref)	24.2	1.00(Ref)
Fetal-exposed	37.3	1.17(0.86-1.59)	32.0	1.25(0.95-1.63)
Childhood-exposed	46.2	1.26(0.94-1.70)	37.3	1.42(1.10-1.84)
Adolescence/adult exposed	61.3	1.27(0.77-2.11)	41.7	1.33(0.86-2.04)
**CDS criteria**				
Non-exposed	19.0	1.00(Ref)	17.3	1.00(Ref)
Fetal-exposed	26.2	1.09(0.78-1.52)	25.4	1.37(1.03-1.82)
Childhood-exposed	34.4	1.18(0.88-1.58)	28.7	1.40(1.09-1.79)
Adolescence/adult exposed	55.1	1.40(0.87-2.26)	38.3	1.58(1.04-2.40)
**IDF criteria**				
Non-exposed	22.7	1.00(Ref)	21.4	1.00(Ref)
Fetal-exposed	31.2	1.23(0.89-1.70)	28.5	1.27(0.96-1.68)
Childhood-exposed	36.9	1.18(0.86-1.60)	34.2	1.52(1.17-1.98)
Adolescence/adult exposed	51.6	1.23(0.74-2.06)	37.8	1.45(0.93-2.26)

Adjusted for age, cohort, sex, education levels, income levels, current smoking and current drinking.
